# Chemerin Levels in COVID-19 Are More Affected by Underlying Diseases than by the Virus Infection Itself

**DOI:** 10.3390/biomedicines12092099

**Published:** 2024-09-14

**Authors:** Vlad Pavel, Pablo Amend, Niklas Schmidtner, Alexander Utrata, Charlotte Birner, Stephan Schmid, Sabrina Krautbauer, Martina Müller, Patricia Mester, Christa Buechler

**Affiliations:** 1 Department of Internal Medicine I, Gastroenterology, Hepatology, Endocrinology, Rheumatology, and Infectious Diseases, University Hospital Regensburg, 93053 Regensburg, Germany; vlad.pavel@klinik.uni-regensburg.de (V.P.); pablo.amend@stud.uni-regensburg.de (P.A.); niklas.schmidtner@stud.uni-regensburg.de (N.S.); alexander.utrata@stud.uni-regensburg.de (A.U.); charlotte.birner@stud.uni-regensburg.de (C.B.); stephan.schmid@klinik.uni-regensburg.de (S.S.); martina.mueller-schilling@klinik.uni-regensburg.de (M.M.); patricia.mester@klinik.uni-regensburg.de (P.M.); 2 Institute of Clinical Chemistry and Laboratory Medicine, University Hospital Regensburg, 93053 Regensburg, Germany; sabrina.krautbauer@klinik.uni-regensburg.de

**Keywords:** COVID-19, liver cirrhosis, ventilation, diabetes, hypertension, intensive care, mortality

## Abstract

Background/Objectives: Chemerin is an adipokine involved in inflammatory and metabolic diseases, and its circulating levels have been associated with inflammatory parameters in various patient cohorts. Severe acute respiratory syndrome coronavirus type 2 (SARS-CoV-2) infection, which causes COVID-19, triggers inflammatory pathways. However, the association between serum chemerin levels and COVID-19 disease severity and outcomes has not been definitively established. Methods: In this study, serum chemerin levels were analyzed in 64 patients with moderate COVID-19 and 60 patients with severe disease. Results: The results showed that serum chemerin levels were comparable between these two groups and slightly higher than in healthy controls. Notably, COVID-19 patients with hypertension exhibited elevated serum chemerin levels, while those with liver cirrhosis had lower levels. When patients with these comorbidities were excluded from the analyses, serum chemerin levels in COVID-19 patients were similar to those in healthy controls. Positive correlations were observed between serum chemerin levels and markers such as alkaline phosphatase, C-reactive protein, eosinophils, and lymphocytes in the entire cohort, as well as in the subgroup excluding patients with hypertension and cirrhosis. Additionally, urinary chemerin levels were comparable between COVID-19 patients and controls, and neither hypertension nor dialysis significantly affected urinary chemerin levels. Both survivors and non-survivors had similar serum and urinary chemerin levels. Conclusions: In conclusion, this study suggests that comorbidities such as arterial hypertension and liver cirrhosis do have a more significant impact on serum chemerin levels than SARS-CoV-2 infection itself.

## 1. Introduction 

Chemerin was first identified as a chemoattractant for innate immune cells [1]. Later, chemerin was described as a novel adipokine that regulates insulin response and adipogenesis [2,3,4]. The observation of low chemerin expression in the squamous cell carcinoma of the skin [5] suggests a role of chemerin in cancer, and various studies have observed pro- and antitumor properties of chemerin [6]. Chemerin also has antimicrobial activity against *Candida albicans*, *Escherichia coli*, and methicillin-resistant *Staphylococcus aureus* [7,8]. 

Serum levels of most adipokines are elevated in obesity, including chemerin [2,3,9]. Higher circulating chemerin levels in hypertensive patients and patients with cardiovascular disease is another consistent finding of various studies [10,11]. Whether circulating chemerin levels are induced in patients with type 2 diabetes has not been finally clarified [2,3,12].

Chemerin bioactivity is regulated by proteolytic cleavage, and the total levels of chemerin in the blood and chemerin bioactivity may not correlate [3]. The angiotensin-converting enzyme (ACE) is a dicarboxypeptidase responsible for C-terminal truncation and the inactivation of chemerin [13]. Whether the monocarboxypeptidase ACE2, which functions as an entry receptor for severe acute respiratory syndrome coronavirus type 2 (SARS-CoV-2), is also involved in chemerin processing is not known [14]. 

Serum chemerin levels were found to be elevated in patients with inflammatory diseases, and positive correlations between serum chemerin and C-reactive protein were observed [15,16,17]. Correspondingly, circulating chemerin was higher in patients with sepsis compared to healthy controls [18,19,20]. However, studies attempting to find associations of chemerin with disease severity and outcome have reported mixed results [18,19,20]. 

SARS-CoV-2 infection has clinical symptoms ranging from asymptomatic states to critical illness [21] and causes severe disease in about 20% of patients, of whom about 5% require intensive care [22]. Risk factors for severe coronavirus disease 2019 (COVID-19) include older age; male gender; obesity; metabolic diseases such as type 2 diabetes and hypertension; immunosuppressive medications; and underlying medical conditions such as cancer, liver cirrhosis, or chronic lung disease [23,24,25,26,27,28,29,30]. 

The identification of circulating biomarkers in patients with severe COVID-19 is of pathophysiological, diagnostic, and prognostic relevance. Systemic chemerin was one of the proteins analyzed in these studies. COVID-19 patients were found to have lower serum chemerin levels compared to healthy controls. In this study, blood chemerin levels were not associated with disease severity or outcomes [31]. In contrast, another study reported higher plasma chemerin levels in non-hospitalized, hospitalized, and intensive care-requiring COVID-19 patients compared to healthy controls, with no significant difference observed among the COVID-19 subgroups. Patients with elevated chemerin levels at admission had an increased risk of mortality from the infection [32]. Finally, it was observed that serum chemerin kinetics during COVID-19 were related to disease severity, with patients having severe COVID-19 showing low chemerin levels on day seven [33]. Patients with a worse prognosis were also reported to have low serum chemerin levels at hospital admission [34]. Additionally, patients with sepsis due to SARS-CoV-2 infection had plasma chemerin levels comparable to those with sepsis from other causes [20]. 

Chemerin is produced by adipose tissues and the liver and has been demonstrated to play a role in the inflammatory process. An increase in serum chemerin has been observed in patients with a range of inflammatory diseases [3,35]. However, the relationship between serum chemerin and SARS-CoV-2 infection remains unclear [36]. The primary objective of this study was to ascertain whether serum chemerin levels are altered in SARS-CoV-2 infection and to identify any associations between serum chemerin levels and underlying diseases with an increased risk of severe illness due to SARS-CoV-2 [23].

## 2. Materials and Methods

### 2.1. Study Cohort

Serum from 124 COVID-19 patients was collected during their hospital stay between 16 April 2020, and 12 January 2024. The serum of this cohort has been employed for the analysis of adiponectin, an adipokine that is almost exclusively produced by adipocytes. The identical cohort was used for this study [37,38]. Urine samples from 84 patients were obtained between 16 April 2020 and 14 June 2021, and all urine samples collected were used for the investigation. 

SARS-CoV-2 infection in these patients was confirmed by polymerase chain reaction. All patients older than 18 years were included in this study. Patients with moderate COVID-19 developed systemic inflammatory response syndrome (SIRS) [39,40] but did not require intensive care admission, while patients with severe COVID-19 developed septic shock and were hospitalized in the intensive care unit (ICU) [39,40]. 

Sixty-four patients with dyspnoea, tachycardia, fever, and fatigue were included in the moderate COVID-19 group [39]. The cohort of patients under consideration is comparable to patients with moderate COVID-19 as defined by the National Institutes of Health [41]. These patients were admitted to the hospital for monitoring purposes but did not require admission to the ICU. Of the 60 patients treated in the ICU, the majority developed acute respiratory distress syndrome (ARDS) and septic shock and exhibited characteristics that correspond to critical illness according to the National Institutes of Health classification of severity [40,41,42,43]. 

The treatment of patients with confirmed or suspected SARS-CoV-2 infection was conducted in accordance with the prevailing clinical guidelines for the management of COVID-19, as endorsed by the European Medicines Agency and the German Federal Joint Committee. The majority of blood samples were obtained in 2021, a period during which the sole approved antiviral medications for the treatment of SARS-CoV-2 infection in Germany were remdesivir and dexamethasone. These agents were also employed for the small number of patients from whom serum samples were collected in 2024.

The control group consisted of 30 males and 26 females, with a mean age of 56 (24–86) years. The gender distribution and age of the controls and COVID-19 patients were similar (*p* > 0.05).

### 2.2. Chemerin ELISA

Serum and urinary chemerin were measured by an enzyme-linked immunosorbent assay (ELISA) (R&D Systems, Wiesbaden, Nordenstadt, Germany; Cat # DY2324). The serum was diluted 1:250 fold for this ELISA. Urine was centrifuged (5 min at 4000 rpm) and used undiluted. Each sample was analyzed in duplicate, and the mean value was calculated and used for further calculations. 

### 2.3. Analysis of Urinary Creatinine

Urinary creatinine was measured by a creatinine parameter assay kit (Cat. No.: KGE005; R&D Systems) in 1:20 fold diluted urine.

### 2.4. Analysis of Urinary Protein Concentration

Pierce™ BCA (bicinchoninic acid assay) Protein Assay (Cat. No.: 23225, Thermo Fisher Scientific, Waltham, MA, USA) was applied for the analysis of urinary protein concentrations using 1:10-diluted urine.

### 2.5. Routine Laboratory Parameters 

The analysis of routine laboratory measures has been described recently [37]. The differential blood count was performed using the impedance/flow cytometry method on a Sysmex instrument supplied by Sysmex Deutschland GmbH, Bornbarch, Germany. The analysis of laboratory parameters was performed at the Institute for Clinical Chemistry and Laboratory Medicine at the University Hospital of Regensburg. Enterococcal resistance to vancomycin was confirmed by PCR analysis of the van A and/or van B genes at the Institute of Clinical Microbiology and Hygiene, University Hospital Regensburg, Regensburg, Germany.

### 2.6. Statistical Analysis

Serum and urine levels of chemerin are displayed as boxplots, with outliers indicated by asterisks or circles. Median, minimum, and maximum values are listed in the tables. The statistical tests used included the Mann–Whitney U test, Kruskal–Wallis test, Spearman’s correlation, and the chi-square test (IBM Corp. Released 2019. IBM SPSS Statistics for Windows, Version 26.0. Armonk, NY, USA: IBM Corp). A *p*-value of <0.05 was considered significant. 

## 3. Results

### 3.1. Serum Chemerin Levels of Controls and Patients with COVID-19 

In this study, the serum of 124 patients with COVID-19 was analyzed. Table 1 provides details of our study cohort. We also measured chemerin in urine in a cohort (n = 84) that only partially overlapped with the serum cohort, and details of these patients are also shown in Table 1.

**Figure 1:**
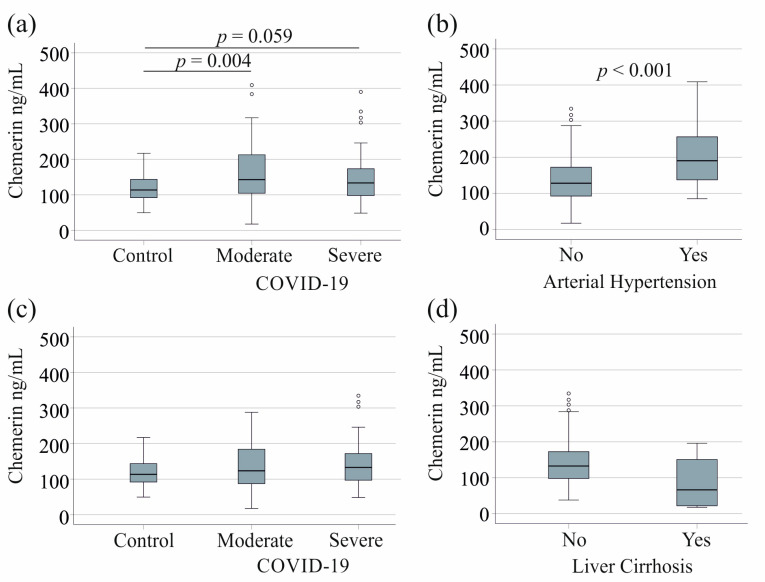
Serum chemerin levels in controls and COVID-19 patients: (**a**) serum chemerin levels of controls, patients with moderate and severe COVID-19; (**b**) serum chemerin levels of COVID-19 patients without and with arterial hypertension; (**c**) serum chemerin levels of controls, patients with moderate and severe COVID-19 after exclusion of patients with hypertension; (**d**) serum chemerin levels of patients with moderate and severe COVID-19 after exclusion of patients with hypertension separated in patients without and with liver cirrhosis.

The 56 controls from whom serum was obtained had a similar gender distribution and age to the patients. Serum chemerin levels were higher in the 64 patients with moderate COVID-19 compared to the 56 controls, and there was a trend toward higher serum chemerin levels in the 60 patients with severe disease compared to the controls (Figure 1a).

In the COVID-19 cohort, serum chemerin levels did not correlate with age (r = 0.063, *p* = 0.490) or BMI (r = 0.097, *p* = 0.370). The 28 patients with diabetes had similar levels to non-diabetic patients (*p* = 0.396). Serum chemerin levels in patients with cardiovascular disease (14 patients, *p* = 0.153) or tumors (15 patients, *p* = 0.780) did not differ from those without these comorbidities. The 24 hypertensive patients had elevated serum chemerin levels (Figure 1b). Excluding the patients with arterial hypertension, serum chemerin levels of controls and patients with moderate and severe COVID-19 disease were similar (Figure 1c). 

The COVID-19 cohort included five patients with liver cirrhosis, who had lower serum chemerin levels [44,45]. This difference was not significant in our cohort (*p* = 0.092 in the whole patient cohort and *p* = 0.143 when patients with hypertension were excluded, Figure 1d). Serum chemerin levels in controls and patients with moderate and severe COVID-19 were still similar (*p* = 0.239) when patients with hypertension and patients with liver cirrhosis were excluded.

### 3.2. Chemerin in Relation to Interventions and Vasopressor Therapy 

Serum chemerin levels in patients on dialysis or mechanical ventilation were similar to those in patients without these interventions. Vasopressor therapy was not associated with changes in chemerin levels. This was true both in the entire cohort and when patients with arterial hypertension and patients with cirrhosis were excluded (Table 2).

### 3.3. Correlation of Serum Chemerin Levels with Laboratory Measures and Adiponectin 

In COVID-19 patients, chemerin positively correlated with C-reactive protein, alkaline phosphatase, eosinophils, and lymphocytes in the entire cohort, as well as after excluding patients with liver cirrhosis and patients with arterial hypertension (Table 3).

Recently, serum adiponectin was measured in this cohort [37]. Serum adiponectin did not correlate with chemerin in the entire cohort (r = −0.071, *p* = 0.432) and when patients with liver cirrhosis and hypertension were excluded (r = −0.092, *p* = 0.377). In patients with moderate COVID-19, who had normal adiponectin levels, adiponectin and chemerin levels were not correlated (r = 0.131, *p* = 0.433). 

### 3.4. Effect of Bacterial and Fungal Infections on Serum Chemerin Levels 

In our cohort, 32 patients developed bacteremia, which was not associated with altered serum chemerin levels (*p* = 0.549). Neither infection with vancomycin-resistant bacteria (n = 10, *p* = 0.474), nor herpes simplex virus reactivation (n = 20, *p* = 0.289), nor fungal infection (n = 23, *p* = 0.409) had any effect on serum chemerin levels.

In the subgroup of patients without hypertension and liver cirrhosis, 27 patients had bacteremia, which also showed no association with altered serum chemerin levels (*p* = 0.506). Even in cases of vancomycin-resistant bacteria (n = 9, *p* = 0.446), herpes simplex virus reactivation (n = 18, *p* = 0.235), or fungal infection (n = 22, *p* = 0.902), there was no change in serum chemerin levels. 

### 3.5. Serum Chemerin Levels and Survival

**Figure 2:**
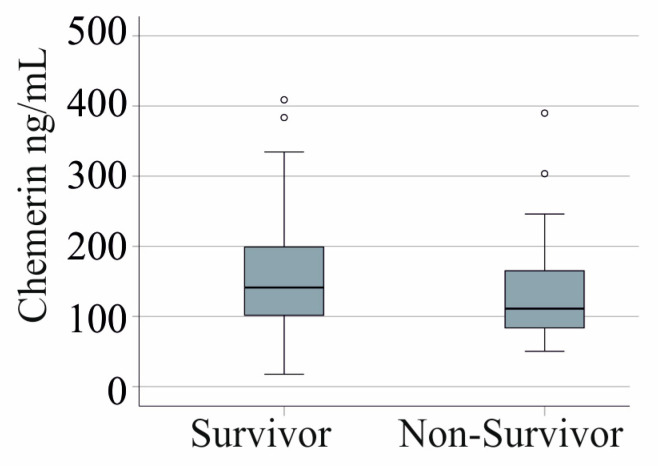
Serum chemerin levels of survivors and non-survivors.

In the entire cohort, 22 patients died. Serum chemerin levels were similar between non-survivors and survivors (*p* = 0.244) (Figure 2). This finding remained consistent in the subgroup of patients without arterial hypertension and liver cirrhosis, where 21 patients did not survive (*p* = 0.319).

### 3.6. Urinary Chemerin 

Urinary chemerin has not been studied in COVID-19 so far. We measured urinary chemerin in 84 patients (51 patients with moderate COVID-19, 33 patients with severe COVID-19, Table 1) and 23 controls (15 women and 8 men, with a mean of 56 (50–81) years old).

Urinary protein is usually normalized to urinary creatinine, which was lower in the patients than in the controls (*p* = 0.013). We also measured total urinary protein, which was much higher in the patients (*p* = 0.008) and was not considered suitable for the normalization of urinary chemerin. 

**Figure 3:**
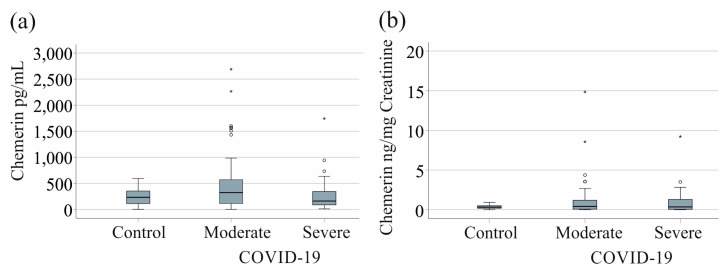
Urinary chemerin levels of controls and COVID-19 patients: (**a**) urinary chemerin levels of controls, patients with moderate and severe COVID-19; (**b**) urinary chemerin normalized to urinary creatinine levels of controls, patients with moderate and severe COVID-19.

Urinary chemerin was similar between controls and patients with moderate and severe COVID-19 (*p* = 0.144), as well as when normalized to urinary creatinine (*p* = 0.510) (Figure 3). 

In the patients, urinary chemerin did not correlate with serum creatinine (r = 0.033, *p* = 0.767), urea (r = 0.011, *p* = 0.922), or albumin (r = 0.207, *p* = 0.095). Chemerin normalized to urinary chemerin was not associated with serum creatinine, urea, or albumin (*p* > 0.05 for all).

Arterial hypertension (13 patients) did not affect urinary chemerin levels (*p* = 0.390) and urinary chemerin/creatinine ratio (*p* = 0.313). Liver cirrhosis (5 patients) did not affect urinary chemerin levels (*p* = 0.917) and chemerin normalized to urinary creatinine (*p* = 0.902).

Dialysis (6 patients), mechanical ventilation (36 patients), and vasoactive therapy (31 patients) were not associated with changes in urinary chemerin levels and urinary chemerin/creatinine ratios (*p* > 0.05). The COVID-19 cohort with available urine samples included 59 patients, in whom serum chemerin was also analyzed. However, serum and urinary chemerin levels were not correlated (*p* = 0.227 for non-normalized, *p* = 0.183 for creatinine-normalized urinary chemerin levels).

**Figure 4:**
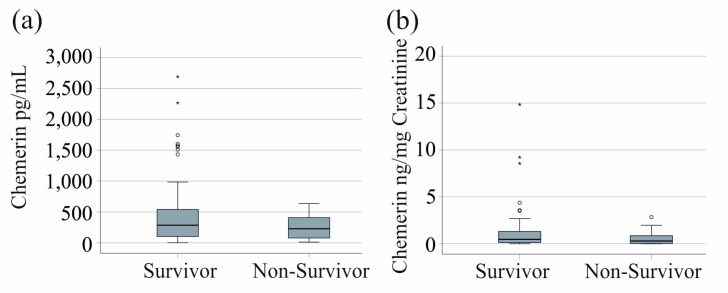
Urinary chemerin levels of survivors and non-survivors: (**a**) urinary chemerin levels of survivors and non-survivors.; (**b**) urinary chemerin normalized to urinary creatinine levels of survivors and non-survivors.

Urinary chemerin levels (*p* = 0.331) and creatinine-normalized urinary chemerin levels (*p* = 0.730) did not differ between survivors and non-survivors (Figure 4).

## 4. Discussion

This study demonstrates that arterial hypertension contributes more significantly to elevated serum chemerin levels in COVID-19 patients than SARS-CoV-2 infection or the severity of COVID-19. Arterial hypertension, known to be associated with increased serum chemerin levels in the general population [10], was also strongly linked to elevated chemerin levels in hypertensive patients with COVID-19.

The studies that have analyzed circulating chemerin levels in SARS-CoV-2 infection have produced inconsistent results [20,31,32,46]. Kukla et al. observed higher chemerin levels in the healthy controls compared to patients with COVID-19. The controls had slightly better liver function, higher serum creatinine, and international normalized ratio, and were slightly younger than the patients. Blood pressure was not documented in this study, and the reason for higher chemerin levels in controls is unclear [31]. Lavis et al. observed higher chemerin levels among COVID-19 patients compared to healthy controls. Patients with liver disease were excluded from the analysis. Patients with COVID-19 were more likely to have hypertension, which may contribute to increased chemerin levels [32]. Hypertension was diagnosed in 41% of COVID-19 patients, and plasma chemerin levels were 165 ng/mL in hypertensive patients and 118 ng/mL in non-hypertensive patients. Plasma chemerin levels in healthy controls were 76 ng/mL and were still lower compared to patients with severe COVID-19. However, patients with mild COVID-19 had 120 ng/mL plasma chemerin, and in this cohort, blood chemerin levels did not differ greatly between mild and severe COVID-19 disease [32]. The patients with the highest chemerin levels in the study published by Gokdemir et al. had the highest blood pressure. In this analysis, the chemerin levels of healthy controls and patients with mild COVID-19 were similar [46]. Amend et al. excluded patients with liver cirrhosis but did not provide information on hypertension. In this study, patients with severe COVID-19 disease had higher plasma chemerin compared to controls [20]. Another study compared severe and non-severe COVID-19 patients with similar prevalence of hypertension and found no difference in serum chemerin levels [33]. Based on these studies, it is reasonable to conclude that arterial hypertension significantly influences circulating chemerin levels. To accurately assess whether serum chemerin levels are altered in COVID-19 patients, this factor must be considered. In our cohort, after excluding patients with arterial hypertension, there was no significant difference in serum chemerin levels between controls and patients with moderate or severe COVID-19.

Chemerin in the blood of various patient cohorts is positively correlated with C-reactive protein [15,47], and this was also observed in those with COVID-19 [32,46]. In our cohort, serum chemerin was positively correlated with C-reactive protein, alkaline phosphatase, eosinophil count, and lymphocytes. These associations were found in the entire cohort and when patients with hypertension and cirrhosis were excluded. 

Adipose tissue is the main source for circulating chemerin levels also in hypertension [10]. Adiponectin is another adipokine almost exclusively released from fat. The serum adiponectin of patients with moderate COVID-19 was normal [37], as were serum chemerin levels, suggesting that the adipose tissue function is not grossly impaired in patients with moderate disease. However, in severe COVID-19 serum, adiponectin was reduced, whereas chemerin levels were still normal. It has been demonstrated that SARS-CoV-2-infected adipocytes exhibit a reduction in the expression of adiponectin [48] while maintaining the normal production of chemerin, as evidenced by the absence of any deviation from the typical serum chemerin levels observed in our patient cohort. However, this hypothesis requires further substantiation through the implementation of appropriate experimental procedures.

Chemerin is an attractant for immune cells, but whether chemerin activity is altered in the serum of patients with COVID-19 has not been studied [3,12]. The eosinophil and lymphocyte counts of our patients with moderate and severe COVID-19 were similar [37], while others reported a decrease in these cells corresponding to higher disease severity [49,50,51]. Correlations of chemerin with lymphocyte and eosinophil counts are therefore unrelated to COVID-19 disease severity. Serum chemerin was also positively correlated with alkaline phosphatase, a marker of cholestatic liver disease [52], with similar levels in patients with moderate and severe COVID-19 [37]. 

The present study did not find any correlations of chemerin with body mass index or higher levels in patients with cardiovascular disease, diabetes, or tumors. Data on chemerin levels in diabetes are inconsistent and both normal and elevated levels have been described [2,12]. Furthermore, correlations between chemerin levels and BMI have not been consistently found [3,9,53]. The number of patients with cardiovascular disease and tumors in our cohort may be too small to detect altered chemerin levels. 

Elevated circulating chemerin levels have also been observed in kidney disease. Current evidence suggests that impaired renal clearance contributes to increased serum chemerin levels, which can be reduced by dialysis [10]. Consistent with this, our dialysis patients exhibited normal serum and urinary chemerin levels.

Statin and metformin therapies are common in Germany but were not documented for our patients [54,55]. Statins protect against SARS-CoV-2 infection and adverse disease outcomes [56,57,58], and they lower the chemerin synthesis of hepatocytes [59]. Metformin therapy was associated with less severe COVID-19 and better survival [60,61], and they lowered chemerin production of hepatocytes and adipocytes [62,63]. Glitazones are insulin-sensitizing drugs with no role in hepatocyte chemerin expression [64] and COVID-19 survival [61,65]. Whether there is a discrepancy in the proportion of patients taking these or analogous medications between the two cohorts has not been established. Nevertheless, the levels of chemerin observed in patients with moderate and severe forms of the disease were comparable, suggesting that significant variations in pharmacological treatment are unlikely.

The vaccination status of the cohorts was not documented, and studies analyzing the association between vaccination and serum chemerin levels have yet to be published. To the best of the authors’ knowledge, urinary chemerin in COVID-19 has never been studied before. Urinary chemerin was not elevated in patients with this infectious disease compared to controls. Furthermore, urinary chemerin was not elevated in hypertension, consistent with the suggestion that adipocyte synthesis is the source of higher serum chemerin in hypertension [10]. 

Urinary chemerin is typically normalized to urinary creatinine [66], which was higher in the controls within our cohort. Therefore, both non-normalized and creatinine-normalized chemerin values were used for calculations, yielding comparable results. Additionally, we found that total urinary protein levels in our patients were significantly elevated compared to healthy controls, making them unsuitable for normalization. Proteinuria is common in COVID-19, and our findings are consistent with previous observations [67].

Arterial hypertension, cardiovascular disease, and diabetes are risk factors for severe COVID-19 and accordingly are more common in patients with severe disease [68,69]. These confounding factors need to be taken into account when analyzing proteins such as chemerin, which are elevated in patients with metabolic disease and especially in hypertension [3,10]. In our cohort, for unknown reasons, hypertension was more common in patients with moderate disease than in those with severe disease. However, the exclusion of these patients showed that SARS-CoV-2 infection was not associated with higher serum chemerin levels.

Liver cirrhosis is also associated with an adverse outcome in patients with severe COVID-19 [25]. Circulating levels of several proteins, including chemerin, are altered in cirrhosis, and this underlying disease needs to be taken into account in future observational studies [44,70,71,72]. 

There are several limitations to this study. Blood and urine were not always collected early after admission to the hospital. We collected spot urine, not morning urine or 24-h urine. The vaccination status and common medications of our patients were not documented. Furthermore, BMI and laboratory measures of controls were not determined. 

## 5. Conclusions

Serum chemerin levels appear to be more influenced by underlying comorbidities, such as arterial hypertension and liver cirrhosis, than by SARS-CoV-2 infection or the severity of COVID-19. Additionally, this study is the first to analyze urinary chemerin levels in COVID-19 patients. However, no significant differences in urinary chemerin levels were observed when compared to healthy controls. Overall, these findings suggest that serum and urinary chemerin levels are not reliable diagnostic markers for COVID-19.

## Figures and Tables

**Table 1:** Characteristics of patients with COVID-19 for analysis of serum and urinary chemerin levels. Data are presented as median, with minimum and maximum values in brackets. Superscript numbers have been added when these laboratory values were not documented for all patients.

Parameter	COVID-19 Serum	COVID-19 Urine
Males/Females	47/77	34/50
Age (years)	60 (22–83)	60 (22–83)
Body mass index kg/m^2^	28.4 (18.4–66.7) ^88^	27.8 (18.4–66.70) ^57^
C-reactive protein mg/L	41 (0–367)	44 (0–367)
Procalcitonin ng/mL	0.16 (0–25) ^106^	0.16 (0–25) ^70^
Lactate dehydrogenase U/L	315 (127–1534) ^100^	282 (136–1534) ^60^
Alkaline phosphatase U/ L	98 (37–743) ^90^	93 (37–972) ^78^
Ferritin ng/mL	823 (32–21976) ^105^	863 (47–5527) ^75^
Interleukin-6 pg/mL	30 (3–1175) ^97^	33 (3–1903) ^70^
Creatinine mg/dL	0.89 (0.16–12.80) ^116^	0.86 (0.16–5.28) ^82^
Urea mg/dL	54 (14–331) ^117^	46 (10–297) ^83^
Albumin g/L	29 (19–43) ^98^	29 (19–43) ^66^
Neutrophils n/nL	6.09 (0.13–24.91)	5.64 (0.13–25.93)
Eosinophils n/nL	0.07 (0–1.19)	0.06 (0–0.88)
Monocytes n/nL	0.65 (0.03–2.52)	0.62 (0.03–2.25)
Lymphocytes n/nL	1.16 (0–75.95)	1.21 (0.12–75.95)
Immature Granulocytes n/nL	0.11 (0–2.92)	0.08 (0–3.06)

**Table 2:** Effect of dialysis, ventilation, and vasopressor therapy on serum chemerin levels. The number of patients is shown as “N”. This was analyzed for the whole cohort and after excluding patients with liver cirrhosis and patients with hypertension.

Intervention/Drug	Whole Cohort		Patients with Hypertension and Liver Cirrhosis Excluded	
	N	*p*-Value	N	*p*-Value
Dialysis	13	0.185	9	0.780
Ventilation	58	0.383	55	0.981
Vasopressor therapy	40	0.332	38	0.125

**Table 3:** Spearman correlation coefficients (r) and *p*-values for the correlation of serum chemerin levels with inflammation measures in the entire cohort of patients with COVID-19 and after excluding patients with liver cirrhosis and patients with hypertension.

Laboratory Measures	Whole Cohort		Patients with Hypertension and Liver Cirrhosis Excluded	
	r	*p*-Value	r	*p*-Value
C-reactive protein mg/L	0.248	0.005	0.315	0.002
Procalcitonin ng/mL	0.135	0.169	0.105	0.338
Lactate dehydrogenase U/L	0.095	0.345	0.105	0.329
Alkaline phosphatase U/L	0.292	0.005	0.365	0.001
Ferritin ng/mL	−0.029	0.771	−0.010	0.926
Interleukin-6 pg/mL	0.073	0.475	0.216	0.057
Creatinine mg/dL	0.158	0.090	0.131	0.215
Urea mg/dL	0.019	0.836	−0.016	0.879
Albumin g/L	0.056	0.581	−0.053	0.644
Neutrophils n/nL	−0.038	0.673	−0.057	0.581
Basophils n/nL	0.082	0.364	0.025	0.813
Eosinophils n/nL	0.374	<0.001	0.373	<0.001
Monocytes n/nL	0.081	0.369	0.094	0.363
Lymphocytes n/nL	0.274	0.002	0.324	0.001
Immature Granulocytes n/nL	−0.055	0.541	−0.045	0.667

## Data Availability

The authors confirm that the data supporting the findings of this study are available within the article. Raw data are available from the corresponding author upon a reasonable request.
